# METTL3 Facilitates Tumor Progression by COL12A1/MAPK Signaling Pathway in Esophageal Squamous Cell Carcinoma

**DOI:** 10.7150/jca.66830

**Published:** 2022-03-28

**Authors:** Jiali Li, Zhenhua Li, Yanzhao Xu, Chao Huang, Baoen Shan

**Affiliations:** 1 Research Center, the Fourth Affiliated Hospital of Hebei Medical University, Shijiazhuang, Hebei, 050011, People's Republic of China; 2 Department of Thoracic Surgery, the Fourth Affiliated Hospital of Hebei Medical University, Shijiazhuang, Hebei, 050011, People's Republic of China

**Keywords:** esophageal squamous cell carcinoma (ESCC), METTL3, COL12A1, MAPK, proliferation, invasion

## Abstract

**Background**: Esophageal squamous cell carcinoma (ESCC) is one of the most common aggressive tumors in the world. m6A modification has been implicated to play an important role in many biological progressions. METTL3 as the main methyltransferase has been found in many cancers, including ESCC. Here, we investigated the underlying mechanism of METTL3 in the development of ESCC.

**Methods**: Quantitative real-time PCR (qRT-PCR), immunohistochemical (IHC) and western blot were used to detect METTL3 expression. To evaluate the function of METTL3, MTS, colony formation, scratch wound healing assay, and transwell and invasion assays were performed. To find out the downstream target of METTL3, mRNA sequencing (mRNA-seq) was conducted. GO and KEGG functional enrichment analyses were carried out to predict possible biological processes and signaling pathways. qRT-PCR and western blot were performed to identify the expression of COL12A1 and the phosphorylation status of RAF, MRK and ERK. Cotransfection of small interfering RNA (for METTL3 silence) with plasmid (for overexpression of COL12A1) and the following gain- and loss-of-function experiments were performed to detect the target gene function of COL12A1 in progression of ESCC mediated by METTL3.

**Results**: Using TCGA database, higher METTL3 expression was found in ESCC tissues. Moreover, we found that METTL3 was significantly increased in ESCC patient tissues compared with normal tissues and correlated with poor prognosis. The expression of METTL3 in ESCC cell lines was assessed. The gain- and loss-of-function indicates that METTL3 promotes cell proliferation, migration and invasion. Additionally, we confirmed that METTL3 can promote the expression of COL12A1 and upregulate the phosphorylation of RAF, MER and ERK, and moreover COL12A1 can restrain siMETTL3-mediated inhibition of proliferation, migration and invasion in ESCC.

**Conclusion**: Our study revealed that METTL3 may have an oncogenic role, facilitating the ESCC progression and metastasis by COL12A1/MAPK signaling pathway.

## Introduction

Esophageal cancer is one of the most aggressive tumors that accounted for sixth dominant cause of cancer-related deaths worldwide 1. Esophageal squamous cell carcinoma (ESCC), as the major histological type of esophageal cancer, accounted for 90% of the cases in the world 2. Despite the great advances in therapeutic options including surgery, radiotherapy, and chemotherapy for ESCC treatment, the overall 5-year survival rate for ESCC patients is still unfavorable 3. Although lots of researches have been made, the exact mechanisms of tumorigenesis and distant metastasis remain unclear. Therefore, more studies are urgently required to investigate the underlying molecular mechanism for developing novel therapeutic approaches for ESCC patients 4.

N6-methyladenosine (m6A) is the most abundant regulation in mRNA, which consisted of m6A methyltransferases, demethylases, and readers 5. In recent years, many research have been reported with respect to the modification and function of m6A, wherein m6A modification was found to play an important role in splicing process, stability, translation efficiency, and nuclear retention of mRNAs and noncoding RNAs 6,7. Methyltransferase-like 3 (METTL3), the main RNA methyltransferase, together with its auxiliary partners METTL14 and WTAP, forms a methyltransferase complex to catalyze the m6A modification 8,9. Alternatively, the demethylases FTO and ALKBH5 remove m6A from mRNA to dynamically regulate the m6A modification 10,11. In addition, m6A readers, including the YTH family proteins, IGF2BPs, and eIF3s, can specifically recognize m6A modification and regulate the splicing, transport, translation, stability, and other functions of the downstream mRNA 12,13. METTL3, the first reported m6A reader, was identified as the main methyltransferase for the methylation process and was further proven to affect the development of many cancers 14-17. However, little researches are focused on the underlying mechanism of METTL3 in the development of ESCC.

In the present study, we elucidate the functional role of METTL3 in ESCC. Our study demonstrates that upregulated METTL3 is associated with proliferation and metastasis of ESCC through COL12A1/MAPK pathway, suggesting METTL3 has an oncogenic role and may indicate a potential biomarker panel for prognostic prediction in ESCC.

## Materials and Methods

### Antibodies

Rabbit anti-METTL3 polyclonal antibody (56339), rabbit anti-MEK1/2 polyclonal antibody (5605), rabbit anti-ERK1/2 polyclonal antibody (5594) and rabbit anti-GAPDH polyclonal antibody (2597) were purchased from proteintech (China); Rabbit anti-COL12A1 antibody (ab121304), rabbit anti-RAF monoclonal antibody (ab181115), rabbit anti-RAF (phospho S259) monoclonal antibody (ab173539), rabbit anti-MEK1/2 (phospho S221+S221) monoclonal antibody (ab278564) and rabbit anti-ERK1/2 (phospho T202+Y204) monoclonal antibody (ab278538) were purchased from Abcam (Cambridge, UK). Mouse anti-β-actin monoclonal antibody (60/11461/81822, Beyotime, China) was purchased from Beyotime (China).

### Clinical specimens

Esophageal cancer tissues and their paired normal tissues were obtained from patients who were diagnosed with esophageal cancer and undergone surgery in the Fourth Hospital of HeBei Medical University between August 2015 and June 2016. The follow-up deadline was June 2021. The enrolled patients were diagnosed to be esophageal cancer according to clinical symptoms, imaging examination and pathological diagnosis, and all the patients had completed clinical parameters, including age, gender, lymph node metastasis, distant metastasis, pathological differentiation, tumor stage, tumor size, TNM staging system and family history. All patients did not undergo the preoperative chemotherapy and radiotherapy. The excluded patients were with other benign as well as malignant tumors, and severe cardiovascular and renal diseases. All patients signed informed consent before using clinical materials. The use of tissues for this study has been proved by the ethics committee of the Fourth Hospital of HeBei Medical University.

### Cell culture

The esophageal cancer cell lines Eca109, TE1, KYSE30, YES2, EC9706, KYSE170 and KYSE150 were obtained from Procell Life Science&Technology Co. (Wuhan, China). Eca109, YES2, EC9706, KYSE170 and KYSE150 were cultured in DMEM medium (GIBCO, USA) containing 10% fetal bovine serum (FBS; Biological Industries, Beit-Haemek, Israel) and 1% penicillin-streptomycin (Solarbio, China) with 5% CO_2_ at 37 °C in a humidified incubator. TE1 and KYSE30 cells were cultured in 1640 medium (GIBCO, USA) containing 10% FBS and 1% penicillin-streptomycin with 5% CO_2_ at 37 °C in a humidified incubator.

### Small interfering RNA transfection

Small interfering RNA (siRNA) oligonucleotides targeting METTL3 (termed as siMETTL3-1 and siMETTL3-2) and negative control RNA oligonucleotides (termed as siControl) were designed and synthesized by RiboBio (Guangzhou, China). The sequence of siRNA is as follows: siMETTL3-1: CAAGTATGTTCACTATGAA and siMETTL3-2: GACTGCTCTTTCCTTAATA. The esophageal cancer cells with 30-50% confluence in 6 wells dishes were transfected with siRNA using ribo FECT ^TM^CP Transfection (RiboBio, China) following the manufacturer's instructions. The knockdown efficiency was evaluated by qRT-PCR and western blots after 40 h.

### Plasmid transfection

Overexpression of METTL3 was conducted by using an expression plasmid (Vigenebio, Shandong) (termed as oeMETTL3) and empty vector (termed as oeVec) was used as the negative control. The esophageal cancer cells were plated in 6 wells dishes at 50-70% confluence transfected with FuGENE® 6 Transfection Reagent (Promega, USA). 48 h later, the efficiency of overexpression was determined by qRT-PCR and western blots.

### Co-transfection

Overexpression of COL12A1 was conducted by using an expression plasmid (Vigenebio, Shandong) (termed as oeCOL12A1) and empty vector (termed as oeVec) was used as the negative control. The esophageal cancer cells with 60-70% confluence in 6 wells dishes were transfected with siMETTL3-1 using Lipofectamine 2000 reagent (Invitrogen, USA) without FBS for 6 h. After that, transfection of oeCOL12A1 or oeVec was performed with FuGENE® 6 Transfection Reagent (Promega, USA) with FBS for the following 24 h.

### RNA extraction and quantitative real-time PCR (qRT-PCR)

Total RNA was isolated from patient tissues and cells by using RNAiso Plus (TaKaRa, Japan) according to the manufacturer's protocol. cDNA was synthesized using MonScript^TM^ RTⅢ All-in-One Mix (Monad Biotech, China) and qRT-PCR for mRNA was performed with MonAmp^TM^ ChemoHS qPCR Mix (Monad Biotech, China) on Step One Plus Real-Time PCR system (Applied Biosystems, USA). GAPDH was used as an internal standard control. Each sample was replicated three times and data was analyzed by comparing Ct values. All PCR primers were purchased from Sangon Biotech (Shanghai, China) and listed in Supplement Table.

### Western blot

Total cellular proteins were lysed by RIPA buffer containing phosphatase inhibitors (SEVEN, China). The protein extractions were harvested and quantified by bicinchoninic acid (BCA) analysis (SEVEN, China). Protein extractions were separated by 12% SDS-PAGE and transferred onto polyvinylidene fluoride (PVDF) membranes (Millipore, USA). After blockade of nonspecific protein binding, membranes were incubated with primary antibodies at 4 °C overnight, and the primary antibodies recognizing METTL3, RAF, RAF (phospho S259), MEK1/2, ERK1/2, β-actin and GAPDH were used by a dilution 1:1000, respectively. The primary antibodies of COL12A1, MEK1/2 (phospho S221+S221) and ERK1/2 (phospho T202+Y204) were used with concentration of 0.1 μg/ml, 0.1 μg/ml and 0.075 μg/ml respectively. The membranes were then incubated with peroxidase (HRP)-conjugated secondary antibody (1:10000, proteintech, China). After washes, signals were detected using a chemiluminescence system (Bio-Rad, USA).

### Immunohistochemistry (IHC)

IHC was performed to compare METTL3 protein expression in esophageal cancer tissues and their paired normal tissues. Praffin sections were treated with xylene and 100% ethanol, followed by decreased concentrations of ethanol. After antigen retrieval, paraffin sections were blocked and stained with anti-METTL3 antibody (56339, proteintech, China) at 4 °C for overnight. After the corresponding secondary antibody was added to the sections for 15 min at 37 °C, DAB (ZSGB-Bio, China) chromogen staining, hematoxylin counterstaining and dehydration were conducted. Immunohistochemical staining was evaluated according to a previously reported scoring method 18. The Scoring combines both representation of the areas and intensities of the stains. In short, the score is the sum of the percentage of positive cells (0, less than 25% positive cells; 1, 26-50% positive cells; 2, 51-75% positive cells, and 3, more than 75% positive cells) and the staining intensity (0, negative; 1, weak; 2, moderate; 3, strong). The sums between 0 and 2 are scored as negative; 3 and 6 are scored as positive. Three experienced pathologists, who were unaware of the clinical data, concurrently reviewed the slides.

### Cell proliferation and colony formation assay

For cell proliferation assay, the transfected cells were seeded into 96-well plates at a density of 2000 cells per well. At 0, 24, 48, 72 and 96 h after seeding, cell viability was assessed by the Cell Titer 96®AQueous One Solution Reagent (Promega, USA) according to the manufacturer's instructions. Briefly, each well was added with 20 μl MTS solution and the plate was incubated at 37 °C for 2 h. The absorbance was measured at 492 nm with a microplate reader (Tecan, USA). For colony formation assay, the transfected cells were seeded into 6-well plates at a density of 1000 cells per well and maintained in DMEM/1640 medium containing 10% FBS for 10 days. After fixed with methanol, the cells were stained with 0.1% crystal violet 30 min and then the colonies were imaged and counted.

### Scratch wound healing assay

Cell migration levels were determined by wound healing assay. Briefly, 5 × 10^5^ cells (per well) were seeded into 6-well culture plate. After incubation for overnight, scratches was performed with the fine end of 200 μl pipette tips (time 0 h). Growing cells for another 24 h, photographs were taken to estimate closure of the gap. The relative distance of cell migration to the scratched area was measured and a healing percentage was calculated. The experiments were repeated in triplicate.

### Transwell and invasion assays

Transwell and invasion assays were conducted using Millicell cell culture inserts (24-well insert, 8-μm pore size, Corning Incorporated, USA) according to the manufacturer's instructions. For migration assay, 4 × 10^4^ cells (per well) in 200 μl serum-free medium were loaded into the bottom of the inserts, then the lower chambers were filled with 500 μl DMEM/1640 medium supplemented with 10% FBS. For invasion assay, Matrigel (BD Biosciences, Franklin Lakes, NJ, USA) was coated into inserts for 4 h before cells were loaded. After 30 h (for migration assay) or 42 h (for invasion assay) of incubation, cells on the underside of the membrane were fixed and stained with 0.5% crystal violet solution. Five random fields were counted in each well under a microscope.

### mRNA-sequencingassay (mRNA-seq)

Total RNAs from the transfected siMETTL3-1 or siControl KYSE150 cells (triplicate for each group) were extracted and purified using RNAiso Plus. Then, mRNA-seq was simultaneously performed on an Illumina Novaseq™ 6000 (LC-Bio Technology, China) following the vendor's recommended protocol. Transcript assembly was examined by using gffcompare software (http://ccb.jhu.edu/software/stringtie/gffcompare.shtml,version:gffcompare-0.9.8.Linux_x86_64). StringTie and ballgown (http://www.bioconductor.org/packages/release/bioc/html/ballgown.html) were used to estimate the different expression of all transcripts.

### Statistical analysis

All statistical analyses were executed with SPSS 21.0 software (SPSS Inc, Chicago, IL, USA). The measurement data were expressed as Mean ± SD, and analyzed by Student's t-test. Chi-square test was used to evaluate the association of expression with the cliniopathological parameters. Overall survival (OS) of patients was estimated by the Kaplan-Meier method, and the log-rank test was used to compare differences between groups. All statistical tests were two-sided, and P value less than or equal to 0.05 was considered statistically significant. At least three independent experiments were performed in every figure.

## Results

### METTL3 was upregulated in human ESCC tissues and correlated with prognosis of ESCC patients

**Figure 1:**
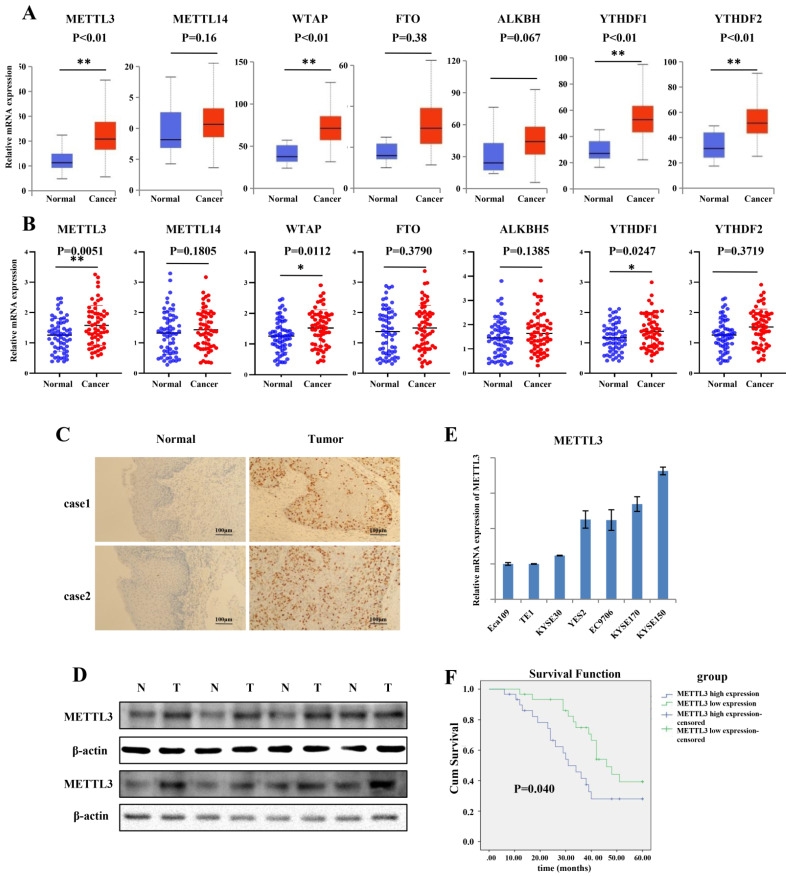
** METTL3 was up-regulated in ESCC patient tissues and associated with poor prognosis.** (A) Catalytic proteins involved in m6a modification were assessed in ESCC tissues downloaded from TCGA data, blue box for normal tissues, n = 11; red box for tumor tissues, n = 184). **P < 0.01. (B) Relative mRNAexpression of catalytic proteins involved in m6a modification was confirmed in the ESCC primary tumor samples and adjacent normal tissues by qRT-PCR (n = 60) normalized against GAPDH. METTL3, WTAP and YTHDF1 were expressed significantly higher in ESCC tissues compared with that in adjacent normal tissues. *P < 0.05, **P < 0.01. (C) Representative image of immunohistochemical staining by METTL3 antibody in two patient tissues at 200× magnification. Scale bars indicated 100 μm. (D) The expression of METTL3 protein in 8 paired ESCC tissues (T) and adjacent normal tissues (N) by western blot. The relative protein levels of METTL3 were normalized against β-actin. (E) mRNA expression level of METTL3 in ESCC cell lines by qRT-PCR. Data are presented as means ± standard deviation. (F) Kaplan-Meier survival curves of OS based on METTL3 mRNA expression in 60 ESCC patients. All patients were divided into two groups based on the median level of METTL3. The log-rank test was used to compare differences. *P < 0.05.

To explore the expression of the major m6A-modifying enzymes in ESCC, including METTL3, METTL14, WTAP, FTO, ALKBH5, YTHDF1 and YTHDF2, we first queried the published clinical data sets TCGA (The Cancer Genome Atlas), and found that mRNA expression of METTL3, WTAP, YTHDF1 and YTHDF2 was significantly increased in ESCC tissues compared to that in normal tissues (Figure 1A). To further validate the results, we obtained 60 paired tumor tissues and adjacent normal tissues of ESCC, and qRT-PCR was used to determine the expression pattern of METTL3, METTL14, WTAP, FTO, ALKBH5, YTHDF1 and YTHDF2. The results indicated that the expression of METTL3, WTAP and YTHDF1 was significantly higher in ESCC tissues than in adjacent normal tissues (Figure 1B). As the major “writer”, together with significant higher expression in ESCC patient tissues, METTL3 was investigated in our experiment. Furthermore, IHC results showed that the expression of METTL3 in tumor tissues was dramatically elevated compared to that in normal tissues (Figure 1C). As expected, the protein level of METTL3 was notably increased in representative ESCC patient tissues compared with adjacent normal tissues by western blot (Figure 1D). We assessed the expression of METTL3 in seven ESCC cell lines (Eca109, TE1, KYSE30, YES2, EC9706, KYSE170 and KYSE150) by qRT-PCR (Figure 1E). The relative mRNA expression of METTL3 was higher in KYSE170 and KYSE150 cell lines, and the mRNA expression of METTL3 was lower in Eca109, TE1 and KYSE30. Based on the mRNA level, in the next gain- and loss-of-function experiment, KYSE150 and KYSE170 cell lines were choose to knockdown METTL3, and TE1 and KYSE30 cell lines were selected to upregulate METTL3 to assess the role that METTL3 may participate in. Kaplan-Meier curves with a log-rank test revealed that ESCC patients with high METTL3 expression exhibited a worse prognosis and shorter survival time compared with that with low METTL3 expression (Figure 1F). To further characterize the correction between METTL3 expression and clinical features, 60 patients were classified into two group on the basis of their median value according to qRT-PCR results (High: n = 30, Low: n = 30). High expression of METTL3 was correlated with lymph node invasion and distant metastasis (Table 1).

### Knockdown of METTL3 inhibited the proliferation and metastatic capacity of ESCC

**Figure 2:**
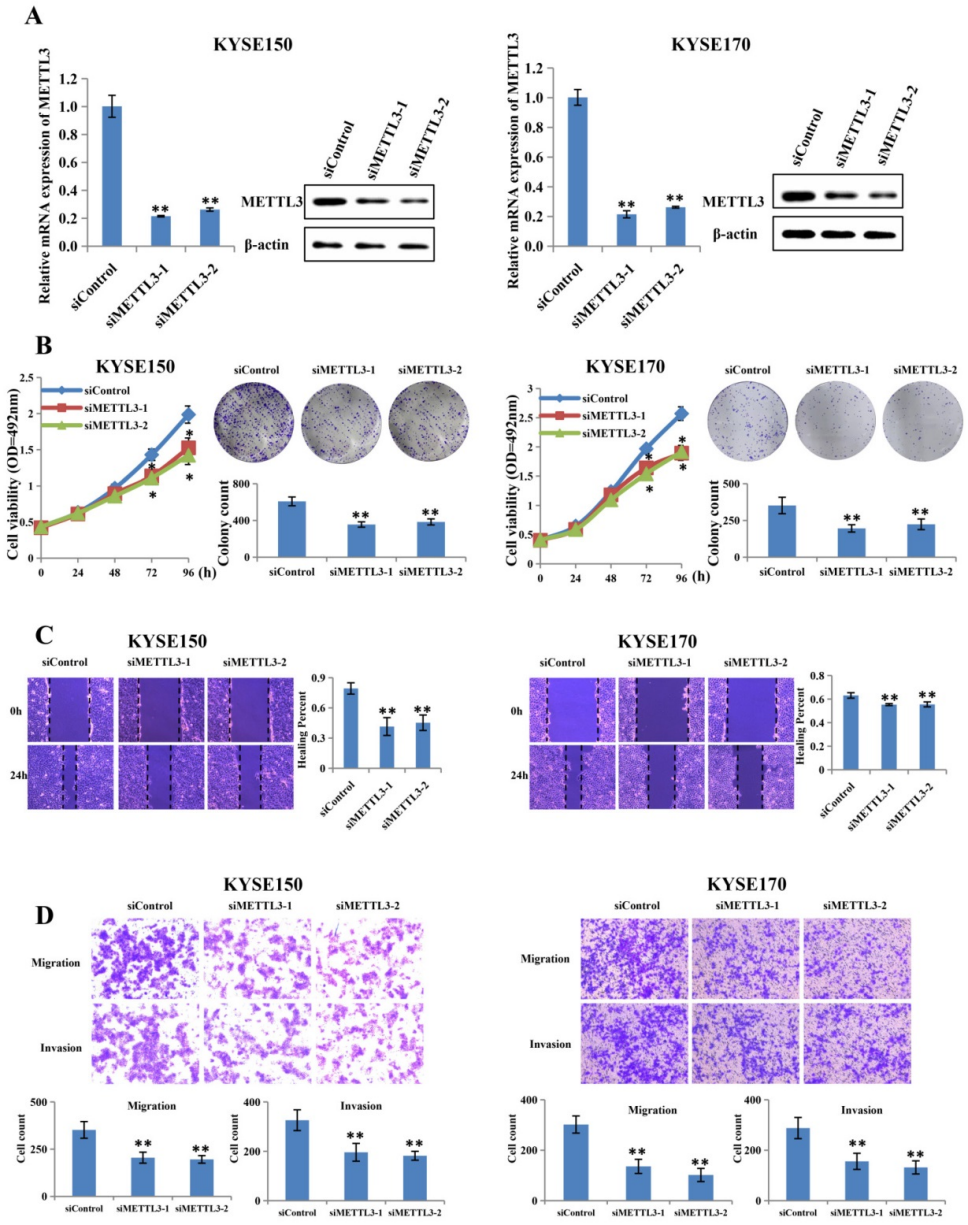
** Silence of METTL3 suppressed proliferation, migration and invasion of ESCC cell lines.** (A) Left and right panel showed the expression level of METTL3 in KYSE150 and KYSE170 cell lines transfected with siMETTL3 or siControl determined by qRT-PCR and western blot. (B) Cell proliferation ability and colony formation ability of KYSE150 and KYSE170 cells transfected with siMETTL3 or siControl were evaluated by MTS assay and colony formation assay. (C-D) Cell migration and invasion abilities of KYSE150 and KYSE170 cells transfected with siMETTL3 or siControl were evaluated by wound healing experiment, transwell migration and matrigel invasion assay. All data are presented as the mean ± SDs (n = 3). *P < 0.05, **P < 0.01.

To investigate whether abnormal METTL3 expression was involved in the proliferation and metastatic capacity of ESCC, KYSE150 and KYSE170 cell lines were transfected with two different siRNAs together with the control RNA (Figure 2A). The knockdown efficiency was assessed by qRT-PCR and western blot, and the results showed that METTL3 expression was knocked down effectively by siRNA in both KYSE150 and KYSE170 cell lines. Proliferation ability was measured by MTS assay and colony formation assay. As expect, the MTS assay showed that METTL3 knockdown led to significantly decreased cell proliferation (Figure 2B). The colony formation assay also displayed that silence of METTL3 decreased the colony formation efficiency (Figure 2B). Scratch wound healing assay and transwell assay were then performed to detect whether METTL3 depletion could affect migration and invasion ability of ESCC. Scratch wound healing assay showed that attenuation of METTL3 expression significantly impeded the migratory ability of ESCC cells as compared to that of the control group (Figure 2C). Correspondingly, transwell migration and matrigel invasion assay confirmed that the invasive ability of ESCC cells was markedly suppressed in response to METTL3 knockdown (Figure 2D).

### Overexpression of METTL3 promoted proliferation and metastatic capacity of ESCC

**Figure 3:**
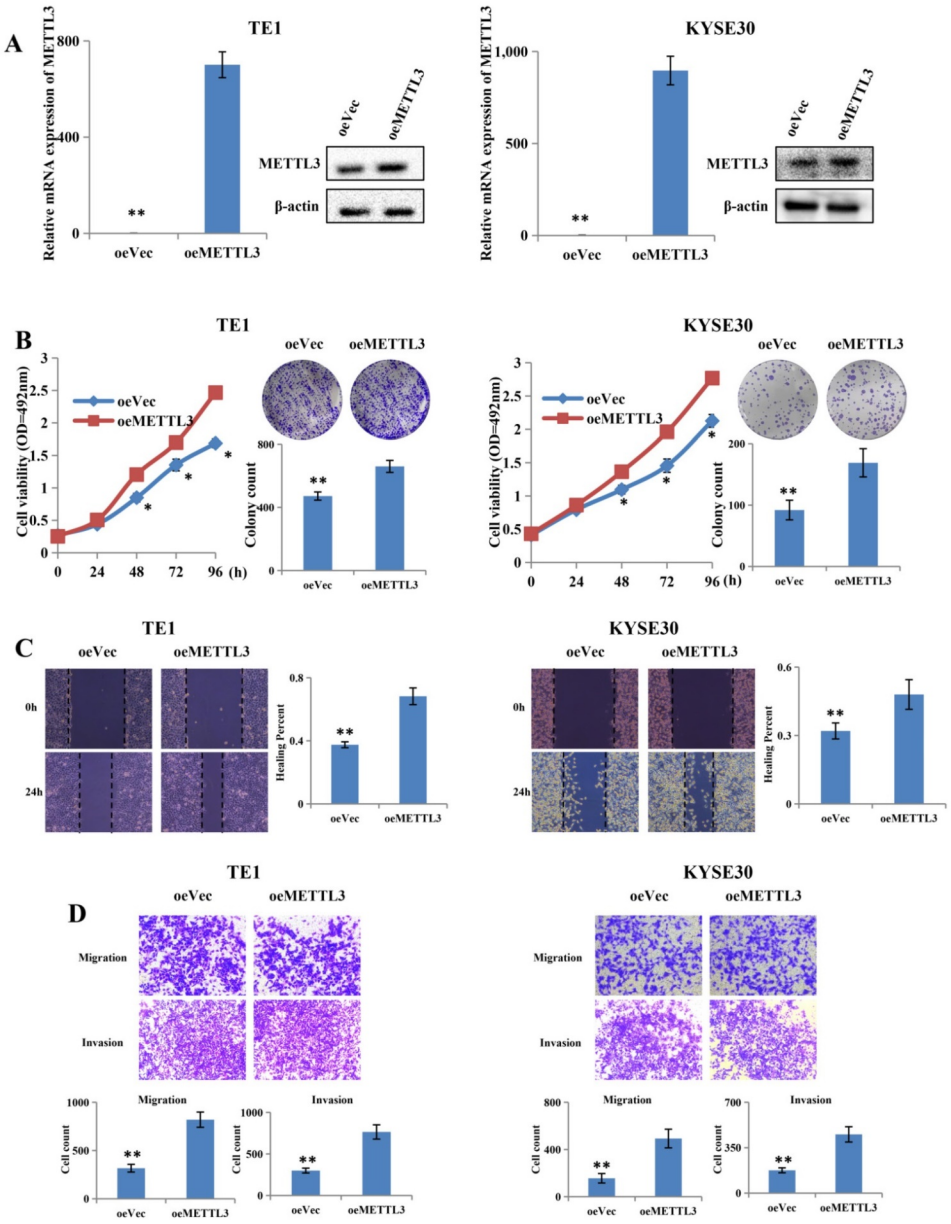
** Overexpression of METTL3 promoted proliferation, migration and invasion of ESCC cell lines.** (A) Left and right panel showed the expression level of METTL3 in TE1 and KYSE30 cells transfected with oeMETTL3 or oeVec plasmid by qRT-PCR and western blot. (B) Cell proliferation ability and colony formation ability of TE1 and KYSE30 cells transfected with oeMETTL3 or oeVec plasmid were evaluated by MTS assay and colony formation assay. (C-D) Cell migration and invasion abilities of TE1 and KYSE30 cells transfected with oeMETTL3 or oeVec plasmid were evaluated by wound healing experiment, transwell migration and matrigel invasion assay. All data are presented as the mean ± SDs (n = 3). *P < 0.05, **P < 0.01.

To further confirm the role of METTL3, two cell lines were selected, including TE1 and KYSE30, for further research, on the basis of their expression pattern. TE1 and KYSE30 cells were transfected with METTL3-overexpressing plasmid and the control plasmid, and then the upregulation efficiency was confirmed at both mRNA and protein levels (Figure 3A). Upregulation of METTL3 in TE1 and KYSE30 cells significantly enhanced their proliferation capacity in MTS assay and colony formation assays (Figure 3B). The scratch wound healing assay showed that METTL3 overexpression accelerates migration and invasion in both TE1 and KYSE30 cells (Figure 3C). In addition, the transwell assay results showed the similar result that upregulation of METTL3 obviously enhances the migration and invasion capacity of ESCC (Figure 3D).

### METTL3 enhanced proliferation and metastasis of ESCC through COL12A1/MAPK signaling pathway

**Figure 4:**
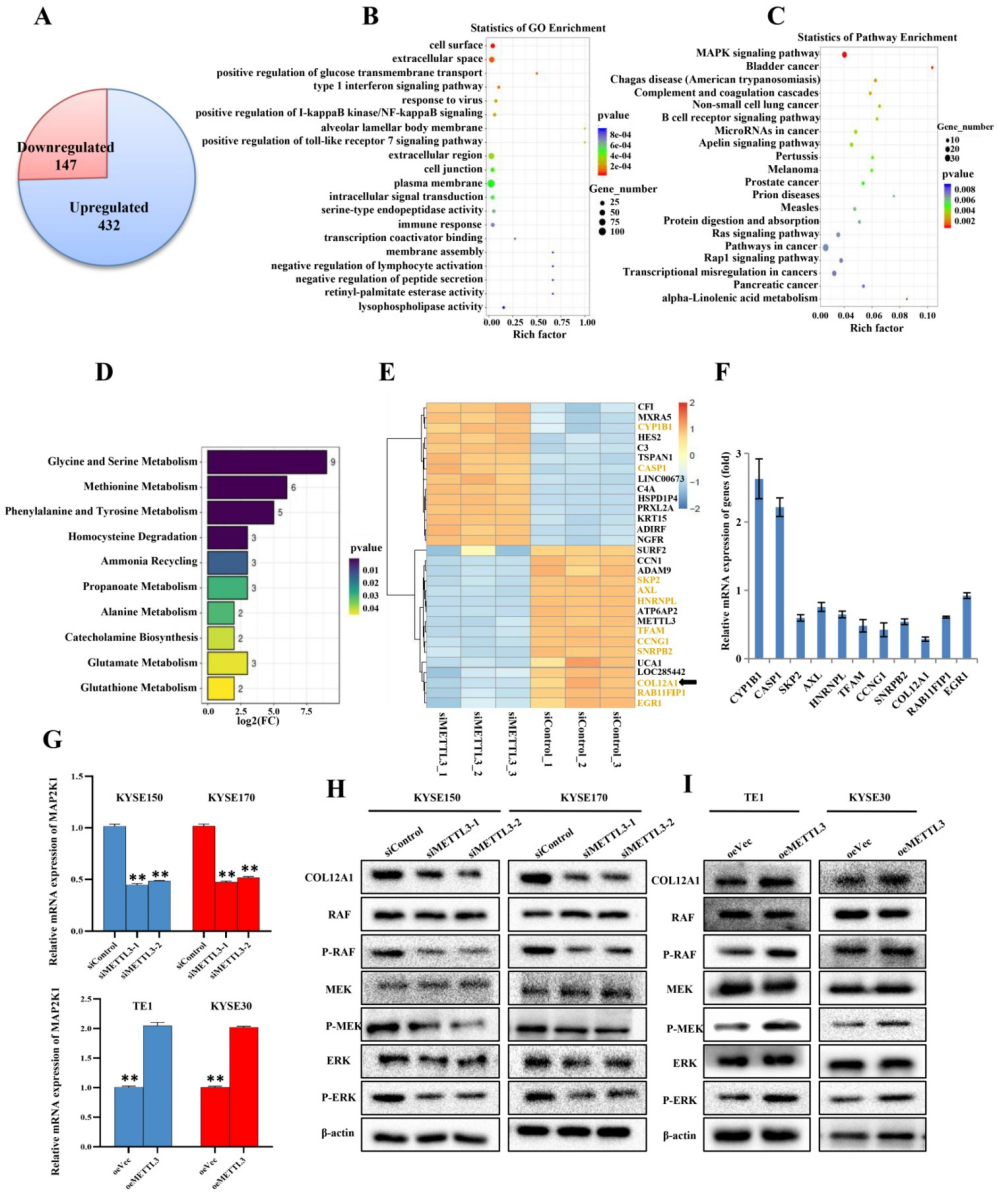
** mRNA-seq assay was performed to validate differentially expressed genes and signaling pathway.** (A) Identification of genes regulated by METTL3 in ESCC using mRNA-Seq assay. (B-C) GO analysis and KEGG pathway analysis predicted possible biological processes and signaling pathways mediated by target mRNAs. (D) GO biological process enrichment analysis of 23 mRNAs involving in MAPK signaling pathway. (E) Heat map of top 30 differentially expressed genes in KYSE150 cells silenced with METTL3. (F) 11 of 30 significantly differentially expressed genes were analyzed by qRT-PCR. (G) Relative level of MAP2K1 was assessed by qRT-PCR in ESCC cell lines with knockdown or overexpression of METTL3. Data are expressed as the mean ± SD. **P<0.01. (H-I) The expression of COL12A1 and phosphorylation status of RAF/MEK/ERK pathway when ESCC cells were transfected with siMETTL3 or oeMETTL3 plasmids was investigated by western blot.

Our previous experiments demonstrated that METTL3 acted as a tumor gene in ESCC. Subsequently, KYSE150 cells with siMETTL3-1 were used to make an in-depth study of downstream target genes and signaling pathways that METTL3 may participate in. We further investigated the mRNA expression by mRNA-seq, which revealed 432 upregulated mRNAs, suggesting that these mRNAs may be negatively regulated by METTL3 (Figure 4A). Correspondingly, 147 mRNAs were downregulated, indicating that these mRNAs may be positively regulated by METTL3 (Figure 4A). GO and KEGG functional enrichment analyses were carried out to predict possible biological processes and signaling pathways mediated by target mRNAs (Figure 4B,C). GO analysis revealed that aberrantly expressed mRNAs were mainly associated with three biological process, including positive regulation of cell surface, extracellular space and positive regulation of glucose transmembrane transport. Pathway analysis detected that these mRNAs were mainly included in mitogen-activated protein kinase (MAPK) cascade. MAPK signaling pathway, covering 23 mRNAs, enriched in 10 different cellular functions, including Glycine and serine metabolism, Methioninemetabolism, Phenylalanine and Tyrosine metabolism (Figure 4D). A heat map was constructed for the siMETTL3-1 group and siControl group and showed top 30 significantly differentially expressed genes between the two groups (Figure 4E). By consulting literatures, 11 of 30 genes, highlighted in Figure 4E, have been reported to participate in the occurrence and development of tumors. We then used qRT-PCR to select and confirm the most significantly related gene COL12A1, the expression of which was decreased to 28.55% in siMETTL3-1 group (Figure 4F). Furthermore, it was reported that COL12A1 positively promoted gastric cancer metastasis via MAPK pathway 19. To further confirm whether METTL3 participates in ESCC progression by COL12A1/RAF/MEK/ERK/MAPK signaling pathway, MAP2K1 (with p<0.01 and log2(fc)=-1.00, suggested by mRNA-seq), which encodes the MEK1 protein, was assessed by qRT-PCR (Figure 4G). METTL3 silence in KYSE150 and KYSE170 cells decreased the expression of MAP2K1, and overexpression of METTL3 in TE1 and KYSE30 cells accelerated the expression of MAP2K1. Western blot was further used to detect the expression of COL12A1 and phosphorylation status of the MAPK signaling pathway. The results showed that METTL3 knockdown in KYSE150 and KYSE170 cells hindered the expression of COL12A1 and RAF/MEK/ERK phosphorylation (Figure 4H), and upregulation of METTL3 in TE1 and KYSE30 cells increased the expression of COL12A1 and the activation of RAF/MEK/ERK (Figure 4I). These findings proved that METTL3 regulated the proliferation and metastasis capacity of ESCC via COL12A1-mediated RAF/MEK/ERK/MAPK signaling pathway.

### COL12A1 counteracted the inhibition effects of siMETTL3-1 on the biology in ESCC

**Figure 5:**
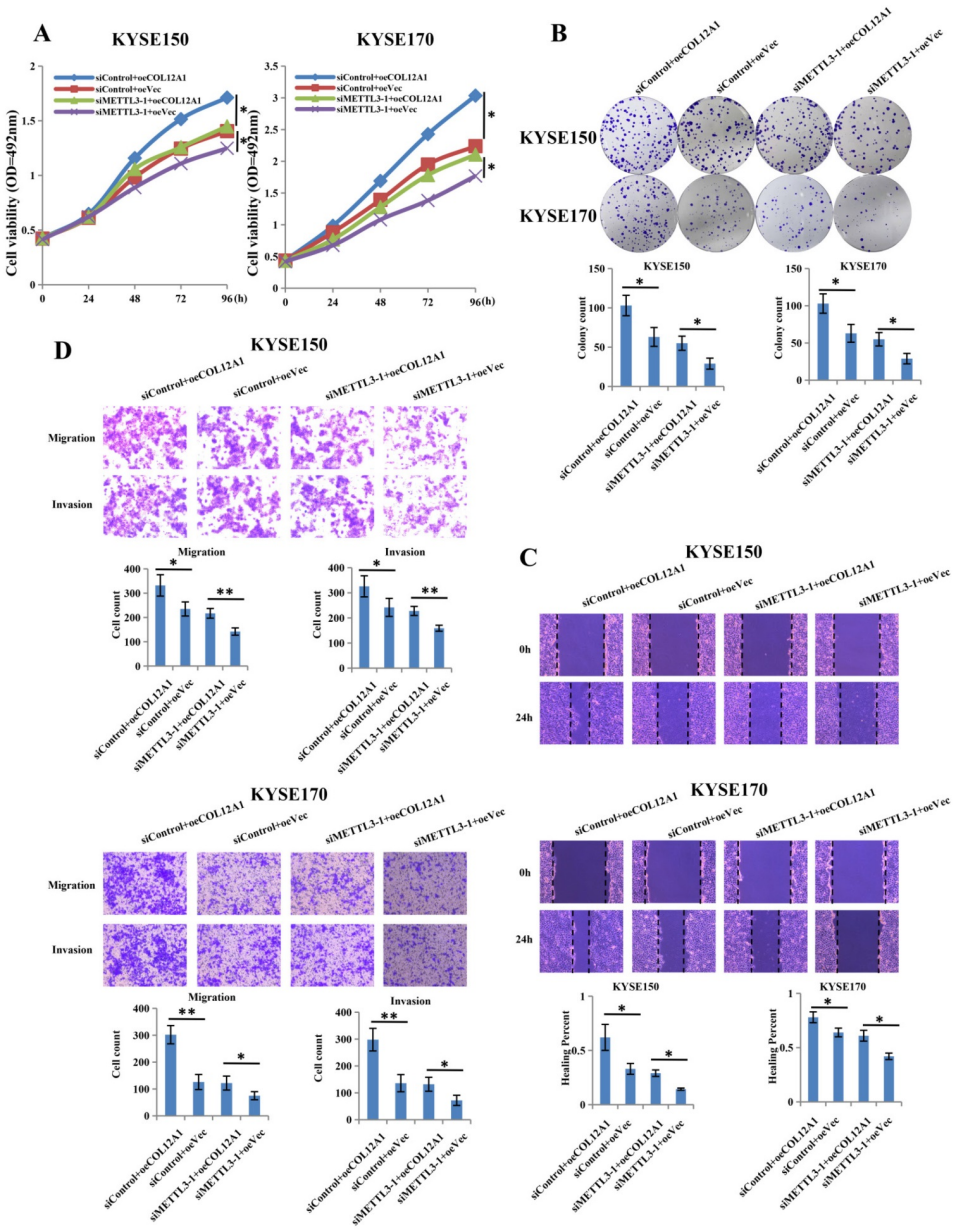
** COL12A1 rescued the effects of siMETTL3-1 on proliferation, migration and invasion of ESCC.** (A-B) siControl+oeCOL12A1 group increased the proliferation and clone formation of ESCC compared with siControl+oeVec group, and siMETTL3-1+oeCOL12A1 group also increased the proliferation and clone formation of ESCC compared with siMETTL3-1+oeVec group. (C-D) siControl+oeCOL12A1 group promoted the migration and invasion of ESCC compared with siControl+oeVec group, and siMETTL3-1+oeCOL12A1 group accelerated the migration and invasion of ESCC compared with siMETTL3-1+oeVec group. *P < 0.05, **P < 0.01.

To investigate the target gene role of COL12A1 in METTL3-promoted proliferation, migration and invasion of ESCC, we constructed overexpression plasmid of COL12A1 to cotransfect with siMETTL3-1 in KYSE150 and KYSE170 cells. Results showed that overexpressed COL12A1 (siControl+oeCOL12A1) group increased cells proliferation and clone formation of KYSE150 and KYSE170 cells compared with control (siControl+oeVec) group, and overexpressed COL12A1 in siMETTL3-1 cells (siMETTL3-1+oeCOL12A1) also increased the proliferation and clone formation of KYSE150 and KYSE170 cells compared with control (siMETTL3-1+oeVec) group (Figures 5A,B). In wound healing and transwell assays, overexpressed COL12A1 (siControl+oeCOL12A1) group promoted the migration and invasion of KYSE150 and KYSE170 cells compared with control (siControl+oeVec) group, and overexpressed COL12A1 in siMETTL3-1 cells (siMETTL3-1+oeCOL12A1) rescued the migration and invasion of KYSE150 and KYSE170 cells compared with control (siMETTL3-1+oeVec) group (Figures 5C,D). These results suggested that COL12A1, positively regulated by METTL3, could be as the target gene, playing a tumor gene in the progression of ESCC.

## Discussion

In recent years, m6A modification has become another epigenetic hot point. First discovered in 1970s, m6A research was revived in 2000s, due to the limitation of research methods 10. Until 2012, with the development of highly specific antibodies and the accessibility of high-throughput sequencing technologies, transcription-wide mapping of m6A sites became feasible, which was a milestone in the field of RNA epitranscriptomics 20,21. Since then, m6A modification was intensely investigated. Installation of m6A is a reversible process regulated from demethylated form to methylated form and vice versa by the balanced activities of m6A “writer” and “eraser” proteins. m6A modification participates in many biological processes in mammals, such as RNA splicing, protein translation, and stem cell renewal 22-24. Many recent studies have partly revealed the underlying mechanisms of m6A modification in cancers. Interestingly, high correlations are found among the expression of different m6A regulators, suggesting extensive crosstalk of the m6A machinery in cancer development 25. Dysregulation of m6A modification and m6A regulators has been proved to play an essential role in kinds of cancer progressions, including cancer stem cell formation, epithelial-mesenchymal transition (EMT), cancer metabolism, and signaling transduction, by regulating the mRNA stability or protein translation of different downstream targets. In breast cancer, ALKBH5 expression is induced in a HIF-dependent manner and overexpression of ALKBH5 reduces m6A modification and stabilizes Nanog mRNA, contributing to breast cancer stem cell formation 26. m6A modification can control cancer metabolism by modulating autophagy by targeting ATG5/7 and regulating pentose phosphate flux by promoting 6PGD translation 27,28. m6A modification also plays an important role in EMT and cancer metastasis by regulating Snail translation in a METTL3- and YTHDF1-dependent manner 29. In addition, m6A modification also regulates multiple signaling pathways, including the AKT, MYC, NF-κB and YAP pathways, to promote cancer growth 30-32. As the major “writer”, METTL3 was frequently investigated in human cancers, either as an oncogene or a tumor suppressor. It was reported that METTL3 was frequently upregulated in colorectal cancer tissues and METTL3/YTHDF2 m6A axis accelerates colorectal carcinogenesis through suppressing YPEL5 16. Increased METTL3 promoted the progression of breast cancer by inhibiting miRNA let-7g 33. In oral squamous cell carcinoma, METTL3 promotes tumorigenesis and metastasis through BMI1 m6A methylation 17. On the other hand, METTL3 as a tumor suppressor also was reported in some cases 34. Lower expression of METTL3 was detected in renal cell carcinoma (RCC) tissues, and higher expression of METTL3 might predict better survival outcome of RCC patients 35. Based on a novel statistical model and the following experimental validation, METTL3 was identified as a tumor suppressor gene in bladder cancer and somatic mutations in METTL3 may promote cancer cell growth 36. However, little researches were focus on the underlying mechanism of METTL3 in the development of ESCC. Here, we identified METTL3 as a pivotal regulator in ESCC, which promoted the development of tumor proliferation and metastasis. We found that high expression of METTL3 was detected in ESCC patient samples and increased METTL3 was significantly associated with poor prognosis. A functional study revealed the essential role of METTL3 in promoting ESCC proliferation, migration and invasion. More importantly, we found that METTL3 regulated tumor progression by COL12A1-mediated MAPK signaling pathway. By GO analysis of RNA-seq, we selected CYP1B1, CASP1, SKP2, AXL, HNRNPL, TFAM, CCNG1, SNRPB2, COL12A1, RAB11FIP1 and EGR1, which were significantly differentially expressed in siMETTL3-1 KYSE150 cells compared to siControl cells, as candidate downstream genes for METTL3 knockdown. Furthermore, we performed qRT-PCR to screen out the most significantly differentially expressed gene COL12A1, which was positively regulated by METTL3. KEGG pathway analysis implied that MAPK pathway hold the most differentially expressed genes. Therefore, we supposed that METTL3 could regulate ESCC progression by COL12A1-mediated MAPK signaling pathway. As for the correlation between COL12A1 and MAPK pathway, Yu et al had reported that COL12A1 promoted gastric cancer metastasis by MAPK pathway, and COL12A1 may be promising targets on anti-cancer treatment in gastric cancer. In addition, we identified the expression of COL12A1 and the phosphorylation status of RAF/MEK/ERK by western blot, and the results showed that knockdown of METTL3 decreased the expression of COL12A1 and the activity of RAF/MEK/ERK. Conversely, and overexpression of METTL3 increased the expression of COL12A1 and the phosphorylation status of RAF/MEK/ERK. To further verify COL12A1 functions as the downstream target gene of METTL3, cotransfection assay of siMETTL3-1 and oeCOL12A1 was performed. Both in siMETTL3-1 and siControl cells, oeCOL12A1 increased the proliferation and clone formation, as well as promoted the migration and invasion in KYSE150 and KYSE170 cells. These suggested that COL12A1, as the downstream gene of METTL3, participated in the progression of ESCC.

## Conclusions

Here, we concluded that METTL3 as an oncogene, promoted both proliferation and metastasis of ESCC by COL12A1/RAF/MEK/ERK/MAPK signaling pathway, suggesting that METTL3 may be a candidate prognostic biomarker for ESCC.

## Supplementary Material

Supplementary table.

## Figures and Tables

**Table 1:** Relevance of Analysis of METTL3 Expression in ESCC Patients.

METTL3 Level								
Characteristics	n	Low		High		χ^2^		P
Age(years)								
<65	29	14	15		0.000		1.000	
>65	31	16	15					
Gender								
Male	46	24	22		0.093		0.760	
Female	14	6	8					
Lymph node metastasis								
Negative	22	16	6		5.813		0.016^*^	
Positive	38	14	24					
Distant metastasis								
Negative	39	24	15		4.689		0.030^*^	
Positive	21	6	15					
Pathological differentiation								
Well	19	11	8		0.308		0.579	
Poor	41	19	22					
Tumor stage								
Stage1,2	25	15	10		1.097		0.295	
Stage3,4	35	15	20					
TNM staging system								
T1+T2	24	14	10		0.625		0.429	
T3+T4	36	16	20					
Tumor size(cm)								
>3	37	16	21		1.128		0.288	
<3	23	14	9					
Family history								
Negative	48	23	25		0.104		0.747	
Positive	12	7	5					
								

NOTE: A chi-square test was used for comparing groups between low and high METTL3 expression. *P < 0.05

## Abbreviations

ESCC: Esophageal squamous cell carcinoma
m6A: N6-methyladenosine
METTL3: Methyltransferase-like 3
MAPK: mitogen-activated protein kinase
