# Supplementary material for: METTL3 Facilitates Tumor Progression by COL12A1/MAPK Signaling Pathway in Esophageal Squamous Cell Carcinoma
Source: J Cancer. 2022 Mar 28;13(6):1972–84. doi: 10.7150/jca.66830 (PMC8990406; doi:10.7150/jca.66830)
Supplement: Supplementary file 1 — Supplementary table. [file jcav13p1972s1.pdf]

Supplement Table. Primer sequences and reaction conditions

Gene	Primer sequence	Annealingtemperature	Product size, bp
METTL3	F: 5’-GAAAGACTATCTCCTGGCACTC-3’<br>R: 5’-GTACCTTTGCTTGAACCGTG-3’	55 ˚C	144bp
METTL14	F: 5’ -TTTCTCTGGTGTGGTTCTGG-3’<br>R: 5’-AAGTCTTAGTCTTCCCAGGATTG-3’	59 ˚C	130bp
WTAP	F: 5’-TTCCCAAGAAGGTTCGATTG-3’<br>R: 5’-TGCAGACTCCTGCTGTTGTT-3’	57 ˚C	192bp
FTO	F: 5’-ACTTGGCTCCCTTATCTGACC-3’<br>R: 5’-TGTGCAGTGTGAGAA AGGCTT-3’	59 ˚C	145bp
ALKBH5	F: 5’-CCCTGCTCTGAAACCCA G-3’<br>R: 5’-GTTCTCTTCCTTGTCCATCTCC-3’	57 ˚C	91bp
YTHDF1	F:5’-ACCTGTCCAGCTATT ACCCG-3’<br>R: 5’-TGGTGAGGTATGGAATCGGAG-3’	57 ˚C	99bp
YTHDF2	F: 5’-TCTGGAAAAGGCTAAGCAGG-3’<br>R: 5’-CTTTTATTTCCCACGACCTTGAC-3’	55 ˚C	146bp
MAP2K1	F: 5’-ATCTGAGGGAGAAGCACAAG-3’<br>R: 5’-CGAAGGAGTTGGCCATGGAG-3’	56 ˚C	135bp
CYP1B1	F: 5’-TTCGGCCACTACTCGGAGC-3’<br>R: 5’-AAGAAGTTGCGCATCATGCT-3’	57 ˚C	68bp
CASP1	F: 5’-GCTTTCTGCTCTTCCACACC-3’<br>R: 5’-TCCTCCACATCACAGGAACA-3’	56 ˚C	119bp
SKP2	F: 5’-TTGCCCTGCAGACTTTGCTA-3’<br>R: 5’-CAGCTGGGTGATGGTCTCTG-3’	57 ˚C	128bp
AXL	F: 5’-ACCTACTCTGGCTCCAGGATG-3’<br>R: 5’-CGCAGGAGAAAGAGGATGTC-3’	56 ˚C	108bp
HNRNPL	F: 5’-TTGTGGCCCTGTCCAGAGAATT-3’<br>R: 5’-GTTTGTGTAGTCCCAAGTATCCTG-3’	57 ˚C	211bp
TFAM	F: 5’-GAGCAGCTAACTCCAAGTCAG-3’<br>R: 5’-TCCGTTTCCTACTAAGCCGAG-3’	59 ˚C	123bp
CCNG1	F: 5’-GTTACCGCTGAGGAGCTGCAGTC-3’<br>R: 5’-GCAGCCATCCTGGATGGATTCAG-3’	60 ˚C	153bp
SNRPB2	F: 5'-TTTAAGGAACTGGGCTCATC-3'<br>R: 5’-TTTGTCAGCAAAAGTTCCAC-3'	51 ˚C	138bp
COL12A1	F: 5’-TATTGTGTTCTTGACTGATGCCTCCTG-3’<br>R: 5’-AGACTTGACCTCATCGCTGTATTGC-3’	59 ˚C	154bp
Rab11FIP1	F: 5’-ATGAACACAACAGCCACCAA-3’<br>R: 5’-CCTTCTTGCTGATGGTCTCC-3’	61 ˚C	99bp
EGR1	F: 5’-GGTCAGTGGCCTAGTGAGC-3’<br>R: 5’-GTGCCGCTGAGTAAATGGGA-3’	58 ˚C	149bp
GAPDH	F: 5’-AATCCCATCACCATCTTCCA-3’<br>R: 5’-TGGACTCCACGACGTACTCA -3’	57 ˚C	82bp
